# SHP2 negatively regulates TGF-β signaling by destabilizing the TGF-β type I receptor

**DOI:** 10.1186/s12964-026-03043-3

**Published:** 2026-07-03

**Authors:** Cen Zhao, Ihor Yakymovych, Mariya Yakymovych, Pengwei Xing, Carl-Henrik Heldin

**Affiliations:** 1 Department of Medical Biochemistry and Microbiology, SciLifeLab, Uppsala University, Box 582, Uppsala, 751 23 Sweden; 2 Department of Immunology, Genetics and Pathology, Uppsala University, Uppsala, 751 08 Sweden

**Keywords:** TGF-β, SHP2, SRC, Protein–protein interaction, Senescence

## Abstract

**Background:**

SRC homology 2 (SH2)-containing protein tyrosine phosphatase 2 (SHP2), acting as a central node of many signaling pathways, has an emerging role in many diseases, including cancer. Transforming growth factor β (TGF-β) affects a wide spectrum of biological processes, including cell proliferation, apoptosis, differentiation and migration, during embryonic development and oncogenesis. TGF-β regulates cell proliferation in a cell-context-dependent manner; loss of TGF-β-mediated growth inhibition is a major characteristic of cancer cells. SHP2 regulates canonical and non-canonical TGF-β signaling in cancer cells and fibroblasts, however, the mechanism by which TGF-β activates SHP2 and the role of SHP2 in TGF-β-mediated growth inhibition has remained unclear.

**Methods:**

The phosphorylation and activation of SHP2 was assessed after TGF-β stimulation of normal and breast cancer cells. The interaction between SHP2 and SRC was determined by co-immunoprecipitation (co-IP) assay. Pharmacological inhibition and gRNA-mediated knockout were applied to assess the role of SHP2 in TGF-β signaling, and RNA-sequencing to identify gene expression patterns in SHP2 knockout cells treated with TGF-β. Functional studies were performed using breast cancer cells to validate the role of SHP2 in TGF-β -mediated growth inhibition.

**Results:**

We report that TGF-β activated SHP2 by promoting its tyrosine phosphorylation by SRC. SHP2 depletion in breast cancer cells reduced ubiquitination and degradation of TβRI by impeding the interaction between TβRI and SMAD7, which is a negative regulator of TβRI. Pharmacological or genetic inhibition of SHP2 facilitated TGF-β-induced SMAD2 phosphorylation and transcriptional responses. Consequently, inhibition of SHP2 profoundly enhanced TGF-β-induced cell growth arrest and senescence, including promotion of TGF-β-induced expression of the cell cycle inhibitor p15 at both gene and protein level.

**Conclusions:**

Our findings uncover a mechanism by which SHP2 is activated by TGF-β in a SRC-dependent manner, and functions in a negative feedback mechanism to regulate TGF-β signaling.

## Background

TGF-β affects a wide spectrum of biological processes, including cell proliferation, apoptosis, differentiation and migration, during embryonic development and oncogenesis [7]. TGF-β can both inhibit and stimulate cell growth, depending on cell type and context. Dysregulation of TGF-β signaling is implicated in many diseases, including cancer [19, 35].

TGF-β exerts its cellular effects by binding to type I and type II dual specificity kinase receptors, TβRI and TβRII, respectively. After ligand binding, the constitutively active TβRII phosphorylates TβRI in the juxta-membrane GS domain; the activated TβRI then activates downstream targets through SMAD-dependent and SMAD-independent mechanisms [11, 37]. In the canonical pathway, receptor-phosphorylated SMAD2 and SMAD3, form complexes with SMAD4, which are translocated into nucleus where they regulate target genes expression [6]. TGF-β also activates non-canonical pathways, including ERK1/2, JNK and p38 MAP-kinases, phosphatidylinositol-3´-kinase (PI3K)-AKT, the tyrosine kinase SRC and NF-κB, in SMAD-independent manners [36]. Phosphorylation of the receptors and downstream transducers plays important roles during the transduction of TGF-β signaling, thus kinases as well as phosphatases are involved in the regulation of TGF-β signaling [26]. The non-receptor tyrosine kinase SRC has been shown to be activated by TGF-β stimulation, promoting fibronectin production, as well as migration of cells [28]. Besides, many protein tyrosine phosphatases (PTPs) are involved in TGF-β signaling. For example, protein tyrosine phosphatase non-receptor 2 (PTPN2) dephosphorylates SMAD4 at Tyr95, promoting the DNA-binding ability of SMAD4, which enhances the growth inhibitory effect of TGF-β in cancer cells [23]. PTPN3 has been shown to impede the interaction between TβRI and the ubiquitin ligase SMURF2, thus stabilizing TβRI and facilitating the phosphorylation of SMAD2/3 independent of its phosphatase activity [32].

SHP2, which is encoded by the *protein tyrosine phosphatase non-receptor type 11* (*PTPN11*) gene, is a cytoplasmatic protein widely expressed in many cell types [3]. SHP2 has two SH2 domains, a protein tyrosine phosphatase domain and a proline-rich C-terminal tail which contains two key phosphorylation sites, Tyr542 and Tyr580 [24]. Even though SHP2 has been shown to be activated by Grb2 binding in a phosphorylation-independent manner, the phosphorylation of SHP2 at Tyr542 has been shown to enhance its phosphatase activity [17]. In fibroblasts, SHP2 is phosphorylated at Tyr542 in response to stimulation by growth factors, such as fibroblast growth factor (FGF) and platelet-derived growth factor (PDGF), creating a binding site for Grb2, evoking activation of the ERK1/2 MAPK pathway [2]. SHP2 has also been reported to affect TGF-β signaling. In lung cancer cells, knockdown of SHP2 partially inhibited TGF-β-induced phosphorylation of SMAD2/3 and epithelial-mesenchymal transition (EMT) [16]. This observation is in contrast to data recently published showing that SHP2 inhibits TGF-β signaling through dephosphorylation of Tyr314 and Tyr434 of the ubiquitin ligase SMURF2 in bladder and lung cancer cell lines [13]. Moreover, the phosphatase activity of SHP2 has been shown to be increased by TGF-β in fibroblasts, leading to dephosphorylation of the inhibitory phosphorylation at Tyr570 of JAK2, thus enhancing JAK2/STAT3 signaling [33]. All these findings highlight the importance of SHP2 in regulating TGF-β activity. Nonetheless, the mechanism by which TGF-β activates SHP2 has remained unclear.

In this study, we demonstrate that TGF-β-activated SRC binds to and phosphorylates SHP2 at Tyr542, promoting its activation. We also report that SHP2 inhibition decreases the binding of the inhibitory SMAD7 to TβRI, thereby impeding the degradation of TβRI, resulting in enhanced TGF-β-induced p15 expression and inhibition of proliferation of breast cancer cells. Taken together, our findings provide novel insights into the regulation of SHP2 by TGF-β and the role of SHP2 in modulating TGF-β signaling in breast cancer.

## Methods

### Cell culture

MCF7, HEK-293 T, HEK-293FT, NMuMG cells were cultured in DMEM medium (Gibco, Life Technologies Ltd, Paisley, UK), supplemented with 10% fetal bovine serum (FBS) (Gibco, Life Technologies Ltd, Paisley, UK), at 37 °C and 5% CO2. 4T1 cells were cultured in RPMI-1640 medium (Gibco, Life Technologies Ltd, Paisley, UK), supplemented with 10% FBS, at 37 °C and 5% CO2.

### Antibodies and reagents

Antibodies against the following proteins were used at the indicated dilutions: HA (sc-805, Santa Cruz, 1:500 for immunoblotting; IB), HA (H3663, Sigma-Aldrich, 1:250 for immunoprecipitation; IP), SRC (2110, Cell Signaling Technology, 1:1000 for IB), p-Y416 SRC (2101, Cell Signaling Technology, 1:1000 for IB), SHP2 (31,259, Cell Signaling Technology, 1:1000 for IB, 1:250 for IP), p-Y542 SHP2 (3751, Cell Signaling Technology, 1:1000 for IB), GFP (A6455, Invitrogen, 1:1000 for IB), pY99 (sc-7020, Santa Cruz, 1:500 for IB), SMAD2 (ab40855, Abcam, 1:1000 for IB), p-S465/467 SMAD2 (generated in rabbits in-house, 1:500 for IB), p15 (sc-271791, Santa Cruz, 1:500 for IB), TβRI (ab235578, Abcam, 1:1000 for IB, 1:250 for IP), Ubiquitin (sc-8017, Santa Cruz, 1:500 for IB), SMAD7 (generated in rabbits in-house, 1:500 for IB, 1:250 for IP), GAPDH (AM4300, Invitrogen, 1:10,000 for IB). Horseradish peroxidase-coupled secondary antibodies were from Jackson ImmunoResearch Laboratories, 1:10,000 dilution.

Protein G Sepharose was from Invitrogen; GFP-Trap Agarose was from Proteintech; PageRuler pre-stained protein ladder was from Thermo Fisher Scientific; Clarity Western enhanced chemiluminescence immunoblotting substrate was from Bio-Rad Laboratories; SRC kinase inhibitor SU6656 and TβRI kinase inhibitor SB505124 were from Sigma-Aldrich; SHP1/2 phosphatase inhibitor NSC-87877 was from Merk Millipore; SHP2 inhibitor SHP099 was from Selleck Chemicals.

### Plasmids

The plasmid pcDNA3-TβRI/HA expressing HA-tagged full-length TβRI was kindly provided by Dr P. ten Dijke (University of Leiden, Netherlands); plasmids expressing SRC (no. 13663) and SRC (K295R, Y527F) mutant (no. 13657) were purchased from Addgene; plasmids expressing GFP-tagged SHP2, RFP-tagged SHP2, RFP-tagged SHP2 (C459S) mutant and GST-tagged SHP2 were kindly provided by Dr Chi-Chuan Lin (Leeds University, UK).

### Generation of TβRI knockout 4T1 cells and SHP2 knockout MCF7 cells

A synthetic AsCas12a crRNA targeting *Tgfbr1* (5’-TGATATGACAACATCAGGGTCTG-3’) was precomplexed with Cas12a protein into ribonucleoprotein (RNP) complexes. RNPs were delivered into 4T1 cells through electroporation using the Neon Transfection System (Thermo Fisher Scientific) with the following settings: 1500 V, 10 ms, 3 pulses, using 5 × 10^5^ cells per 10 µl tip). Single cells from the edited cell pool were sorted into 96-well plates using the SH800S cell sorter (Sony Biotechnology) to obtain clonal lines.

A synthetic gRNA targeting *PTPN11* (5’-CACCGAAGAGTTACATTGCCACACA-3’) was cloned into lentiCRISPR v2 and co-transfected into HEK-293FT cells with pCMV8.91 and pMD2.G to produce lentiviral particles. The medium was collected and 5% PEG8000 in 0.15 M NaCl was added to precipitate viral particles overnight at 4 °C. Viral particles were collected by centrifuging at 4100 × g for 15 min and used to transduce MCF7 cells, followed by selection with 1 µg/mL puromycin; cells were then seeded into 96-well plates to establish a stable SHP2 knockout clonal cell line.

### Transient transfection

Cells were seeded in cell culture dishes or plates. Transient transfections were performed using FuGENE® HD Transfection Reagent kit (Promega, Madison, USA), according to the protocol of the manufacturer.

### Immunoblotting (IB) analysis

Cells were lysed in RIPA lysis buffer (20 mM Tris, pH 7.5, 1% NP-40, 0.5% sodium deoxycholate (DOC), 0.1% sodium dodecyl sulfate (SDS), 5 mM EDTA, 150 mM NaCl) containing 2 mM Na_3_VO_4_, 2 mM NaF, 1 mM β-glycerophosphate and protease inhibitors (Roche Diagnostics, Scandinavia AB, Bromma, Sweden), and centrifuged at 16,000 × g for 15 min. The proteins in the supernatant were separated by SDS–polyacrylamide gel electrophoresis (SDS-PAGE) using 10% or 12% polyacrylamide gels and transferred to nitrocellulose membrane (Amersham Protran). Membranes were blocked by 5% bovine serum albumin (BSA) dissolved in TBST (TBS with 0.1% Tween 20) for 1 h and then incubated with indicated antibodies; protein bands were visualized by enhanced chemiluminescence.

### Co-immunoprecipitation (co-IP) assay

Cells were lysed in IP lysis buffer (20 mM Tris, pH 8.0, 1% Triton X-100, 10% glycerol, 2 mM EDTA, 137 mM NaCl), followed by precleaning with Protein G Sepharose for 2 h; lysates were then incubated with indicated antibody overnight at 4 °C. The next day, the lysate-antibody mix was incubated with Protein G Sepharose at 4 °C for 2 h, after which the beads were washed 4 times with co-IP lysis buffer and one time with Tris-buffered saline (TBS). Proteins were eluted in 2X Laemmli SDS-sample buffer (Bio-Rad Laboratories) and heated at 95 °C for 5 min.

### Proliferation assay

Cells were seeded in 96-well plates, starved overnight, and then treated with TGF-β (5 ng/ml) and indicated inhibitors for 3 days. The number of cells was determined on day 0 and day 3 using the MTT assay.

### Quantitative RT-PCR

RNA was extracted with total RNA purification kit (NORGEN BIOTEK), followed by reverse transcription with the High-Capacity cDNA Reverse Transcription Kit (Applied Biosystems). Quantitative PCR was performed with CFX96 system using 2 × qPCRBIO SyGreen Mix with Fluorescein (PCRBIOSYSTEMS). The relative mRNA expression levels of target genes were calculated with the ΔC_t_ method, using GAPDH as a reference gene. Primer sequences: *GAPDH* Forward, 5’-GGAGTCAACGGATTTGGTCGTA −3’; *GAPDH* Reverse, 5’- GGCAACAATATCCACTTTACCA-3’; *CDKN2B* Forward, 5’-CTAGTGGAGAAGGTGCGACA-3’; *CDKN2B* Reverse, 5’-CATCATCATGACCTGGATCGC-3’.

### In vitro kinase assays

The in vitro kinase assays were performed with GST-tagged SHP2 expressed in bacteria and commercial recombinant His-tagged SRC (Thermo Fisher Scientific). The proteins were mixed in kinase buffer (50 mM HEPES, pH 7.5, 10 mM MgCl_2_, 0.01% Triton X-100), and the reaction was initiated by adding 5 mM ATP. After incubation at 30 °C for 30 min, the reaction was terminated by adding SDS sample buffer and heating at 95 °C for 5 min. The phosphorylation of SHP2 was determined by immunoblotting.

### Phosphatase assays

The phosphatase assays were performed with SHP2 or GFP-tagged SHP2 expressed in HEK293 cells. The proteins were mixed in phosphatase dilution buffer (50 μM imidazole, pH 7.2, 0.2% 2-mercaptoethanol, 65 ng/μl BSA). The reaction was initiated by adding 250 mM *p*-nitrophenyl phosphate (pNPP). After incubation at 37 °C for 2 h, the absorbance of the product was measured at 405 nm.

### Luciferase assay

Cells were transfected with pCAGA12-MLP-Luc and pCMV-β-galatosidase for 24 h. Additional constructs were included in transfections, as indicated. Cells were then treated or not with TGF-β (5 ng/ml) and indicated inhibitors for 2 days. The luciferase activity was measured by Luciferase Assay System kit (Promega, Madison, USA), according to the protocol of the manufacturer.

### RNA sequencing (RNA-Seq)

Total RNA of each sample was quantified by NanoDrop Lite Plus (Thermo Scientific). A total of 200 ng RNA was used for library preparation. Next-generation sequencing library preparations were constructed according to manufacturer’s instructions on the NovaSeq 6000 platform (Illumina Inc., San Diego, CA, USA). Paired-end RNA sequencing data was processed using the nf-core/rnaseq (v3.21.0) [20] pipeline. Briefly, raw FASTQ files were quality-trimmed with Trim Galore (v0.6.10) [9] and aligned to the human reference genome (hg38) using STAR (v2.7.0) [8]. The resulting BAM files were sorted and indexed with samtools (v1.16.1) [15]. Gene-level quantification was performed using Salmon [21], and expression values were normalized as transcripts per million (TPM). Differential expression analysis was conducted with DESeq2 (v1.48.2) [18]. Genes exhibiting an absolute fold change greater than 1.5 (|FoldChange|> 1.5) and a false discovery rate (FDR) below 0.05 were considered significantly differentially expressed. Differentially expressed genes identified from transcriptomic datasets were subjected to Gene Ontology enrichment analysis using the enrichGO function from the clusterProfiler (v4.0) R package [27]. Significant GO terms were defined at an FDR < 0.05.

## Results

### TGF*-*β stimulation induces phosphorylation of SHP2

**Fig. 1:**
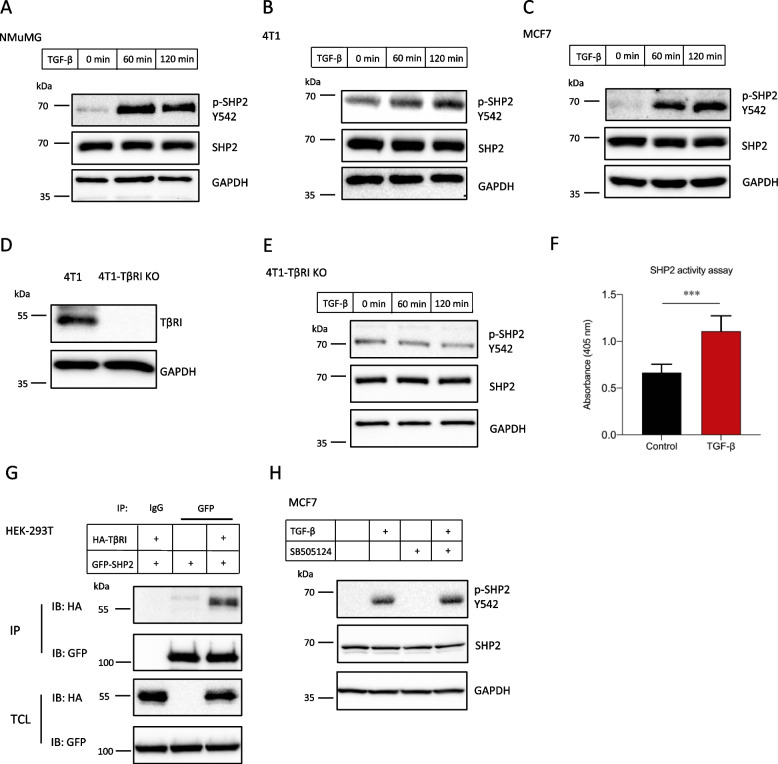
TGF-β stimulation induces phosphorylation of SHP2. **A**-**C** NMuMG (**A**), 4T1 (**B**) and MCF7 (**C**) cells were treated or not with TGF-β (5 ng/ml) for the indicated time periods. Cell lysates were subjected to immunoblotting (IB). **D** Wild-type and TβRI knockout 4T1 cells were subjected to IB to verify knockout of TβRI. **E** TβRI knockout 4T1 cells were treated or not with TGF-β (5 ng/ml) for the indicated time periods. Cell lysates were subjected to IB. **F** MCF7 cells were treated with TGF-β (5 ng/ml) for 2 h. Cell lysates were subjected to immunoprecipitation (IP) with a SHP2 antibody, and the SHP2 activity was assessed with an in vitro phosphatase assay. **G** Lysates of HEK-293 T cells transfected with indicated plasmids were subjected to IP with control IgG or a GFP antibody and then to IB with antibodies against HA and GFP. **H** MCF7 cells were treated with 10 μM SB505124 and TGF-β (5 ng/ml) for 2 h. Cell lysates were subjected to IB

We investigated if TGF-β stimulates the phosphorylation and activation of SHP2 in the mouse mammary gland epithelial cell line NMuMG (Fig. 1A), the mouse breast cancer cell line 4T1 (Fig. 1B) and the human breast cancer cell line MCF7 (Fig. 1C); treatment with TGF-β for 120 min, followed by immunoblotting using an antiserum against phosphorylated Tyr542 in SHP2, revealed that TGF-β induced SHP2 phosphorylation in all three cell lines. Furthermore, we knocked out TβRI in 4T1 cells and investigated the effect of TGF-β-induced SHP2 phosphorylation (Fig. 1D); no increased SHP2 phosphorylation was detected in cells depleted of TβRI (Fig. 1E). Given that Tyr542 phosphorylation of SHP2 promotes its phosphatase activity [38], we assessed SHP2 activity following TGF-β stimulation. Using an in vitro phosphatase assay, we found an increase in SHP2 activity in response to TGF-β treatment (Fig. 1F).

To investigate whether SHP2 interacts with TβRI, we co-expressed HA-tagged TβRI and GFP-tagged SHP2 in HEK-293 T cells; co-immunoprecipitation assays revealed that SHP2 interacted with TβRI (Fig. 1G). To further assess the role of TβRI kinase activity in SHP2 phosphorylation, we treated MCF7 cells with the TβRI kinase inhibitor SB505124 before stimulation by TGF-β and then determined the phosphorylation of SHP2 on Y542. Interestingly, treatment with the TβRI kinase inhibitor SB505124 did not prevent Tyr542 phosphorylation of SHP2 (Fig. 1H). These findings suggest that TGF-β induced SHP2 phosphorylation at Tyr542 in a manner that is independent of the kinase activity of TβRI.

### TGF*-*β stimulation enhances the binding of SRC to SHP2

**Fig. 2:**
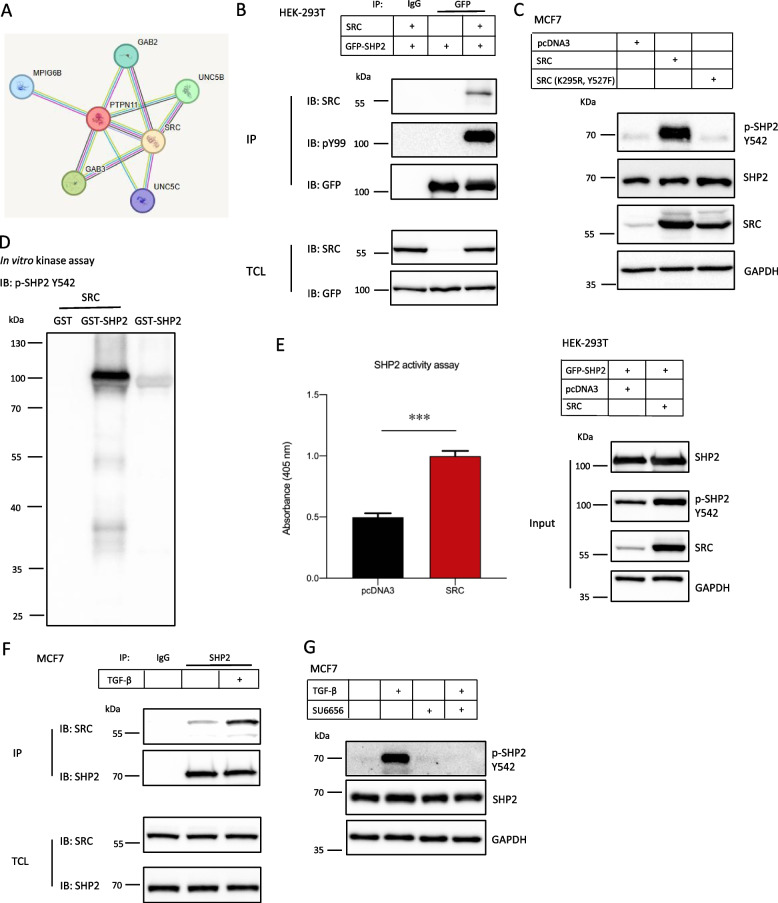
TGF-β enhances the binding of SRC to SHP2.** A** The interaction network of SHP2 shown in the STRING database, version 12.0, interaction confidence ≥ 0.7.** B** Lysates of HEK-293 T cells transfected with indicated plasmids were subjected to IP with control IgG or a GFP antibody, and then to IB with antibodies against SRC, pY99 and GFP. **C** MCF7 cells were transfected with wild-type SRC or mutant SRC (K295R, Y527F) plasmids. Cell lysates were subjected to IB. **D** Purified GST-SHP2 was incubated with recombinant SRC in an in vitro kinase assay and then subjected to IB with a p-SHP2 antibody. **E** Lysates of HEK-293 T cells transfected with SRC and GFP-SHP2 plasmids were subjected to IP with an a SHP2 antibody to assess SHP2 activity using an in vitro phosphatase assay (left), or to analyze expression and phosphorylation of SHP2 and SRC by IB (right). **F** Lysates of MCF7 cells treated with TGF-β (5 ng/ml) for 2 h were subjected to IP with control IgG or anti-SHP2 antibody and then to IB with antibodies against SRC and SHP2. **G** MCF7 cells were treated with 5 μM SU6656 and TGF-β (5 ng/ml) for 2 h. Cell lysates were subjected to IB

Our earlier work revealed that TGF-β activates the tyrosine kinase SRC and promotes its association with TβRI [28]. SHP2 has been shown to collaborate with SRC to modulate breast cancer cell migration and proliferation [22, 29]. Analysis of data from the STRING database (Fig. 2A) suggested that SHP2 interacts with SRC, which was subsequently experimentally verified by co-immunoprecipitation analysis of HEK-293 T cells transfected with SRC and GFP-SHP2 (Fig. 2B).

The role of SRC in transmitting cellular signals involves tyrosine phosphorylation of numerous proteins. We observed that SRC overexpression caused SHP2 phosphorylation at tyrosine residues, as indicated by immunoblotting using antibodies against phospho-tyrosine (pY99) (Fig. 2B) or phosphorylated SHP2 (Y542) (Fig. 2C). Whereas SHP2 was phosphorylated at Tyr542 upon overexpression of wild-type SRC, this was not the case after overexpression of a kinase-dead SRC mutant (Fig. 2C). Using an in vitro kinase assay, we confirmed that SRC directly phosphorylated SHP2 at Tyr542 (Fig. 2D). To explore whether SRC-induced SHP2 phosphorylation affects the enzymatic activity of SHP2, we performed a phosphatase assay; we found an increase in SHP2 activity upon SRC overexpression in HEK-293 T cells (Fig. 2E). Moreover, TGF-β stimulation increased the interaction between SHP2 and SRC, as determined by co-immunoprecipitation in MCF7 cells (Fig. 2F). To further investigate the role of SRC in TGF-β-induced SHP2 phosphorylation, we treated MCF7 cells with the SRC inhibitor SU6656 and found that the SRC inhibitor impeded SHP2 phosphorylation caused by TGF-β stimulation (Fig. 2G). These findings suggest that TGF-β stimulation enhances the binding of SRC to SHP2, thus promoting SRC phosphorylation of SHP2 at Tyr542 and its activation.

### SHP2 interacts with TβRI and modulates its tyrosine phosphorylation

**Fig. 3:**
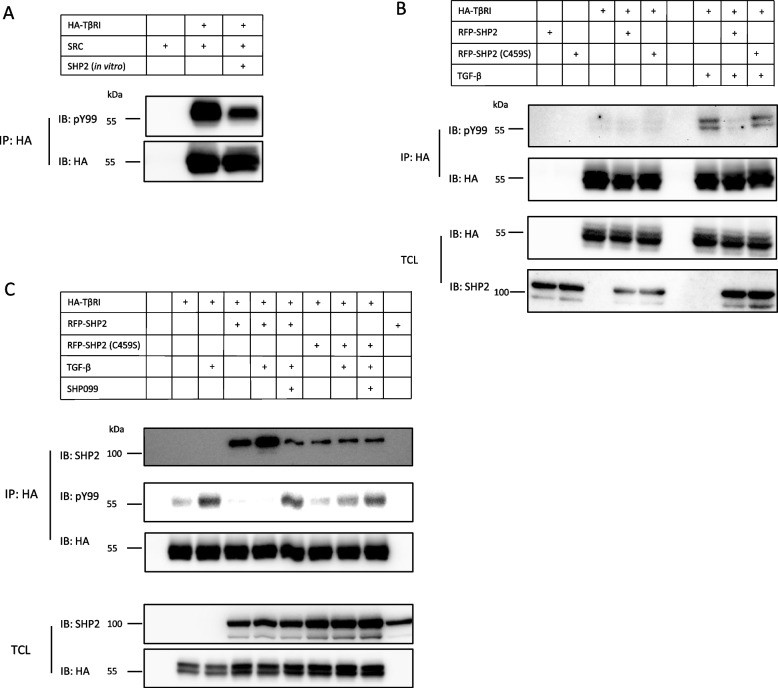
SHP2 interacts with TβRI and modulates its tyrosine phosphorylation. **A** Lysates of HEK-293 T cells transfected with indicated plasmids were subjected to IP with an HA antibody and incubated with recombinant SHP2, and then to IB with antibodies against pY99 and HA. **B** Lysates of HEK-293 T cells transfected with indicated plasmids and treated with TGF-β (5 ng/ml) for 30 min, were subjected to IP with an HA antibody and then to IB with antibodies against pY99, HA and SHP2. **C** Lysates of HEK-293 T cells transfected with indicated plasmids and treated or not with SHP099 (10 μM) and TGF-β (5 ng/ml) for 30 min, were subjected to IP with an HA antibody and then to IB with antibodies against SHP2, pY99 and HA

Given that SHP2 is a tyrosine phosphatase, we investigated whether SHP2 interaction with TβRI leads to decreased TβRI tyrosine phosphorylation. To examine this possibility, we employed an in vitro assay. HA-TβRI and SRC were co-expressed in HEK-293 T cells, followed by immunoprecipitation of HA-TbRI and incubation with purified SHP2. Immunoblotting with a pY99 antibody revealed that incubation with SHP2 significantly reduced the SRC-induced tyrosine phosphorylation of immunoprecipitated TβRI in vitro (Fig. 3A).

We then examined if SHP2 dephosphorylates TbRI also in cells. HEK-293 T cells were co-transfected, as indicated, with HA-TβRI, RFP-tagged wild-type SHP2, and its inactive mutant, RFP-SHP2(C495S). The TGF-β-induced increase in TβRI tyrosine phosphorylation was substantially lower in cells co-expressing wild-type SHP2 compared to those expressing the enzymatically deficient SHP2 (C495S) mutant (Fig. 3B).

Using co-immunoprecipitation, we found that TGF-β stimulation enhanced the interaction between TβRI and wild-type SHP2; whereas the SHP2 (C495S) mutant also interacted with TbRI, the interaction was not enhanced by TGF-b stimulation, suggesting that the enzymatic activity of SHP2 promoted the TGF-b-induced interaction (Fig. 3C). Consistent with this notion, treatment with the SHP2 inhibitor SHP099 abolished the TGF-β-induced interaction between TβRI and SHP2 (Fig. 3C). Moreover, the tyrosine phosphorylation of TbRI was enhanced after expression of the enzymatically deficient SHP2 mutant, or by treatment with a SHP2 inhibitor (Fig. 3C).

These results demonstrate that SHP2 interacts with TβRI and negatively regulates its tyrosine phosphorylation.

### SHP2 inhibitsTGF*-*β signaling

**Fig. 4:**
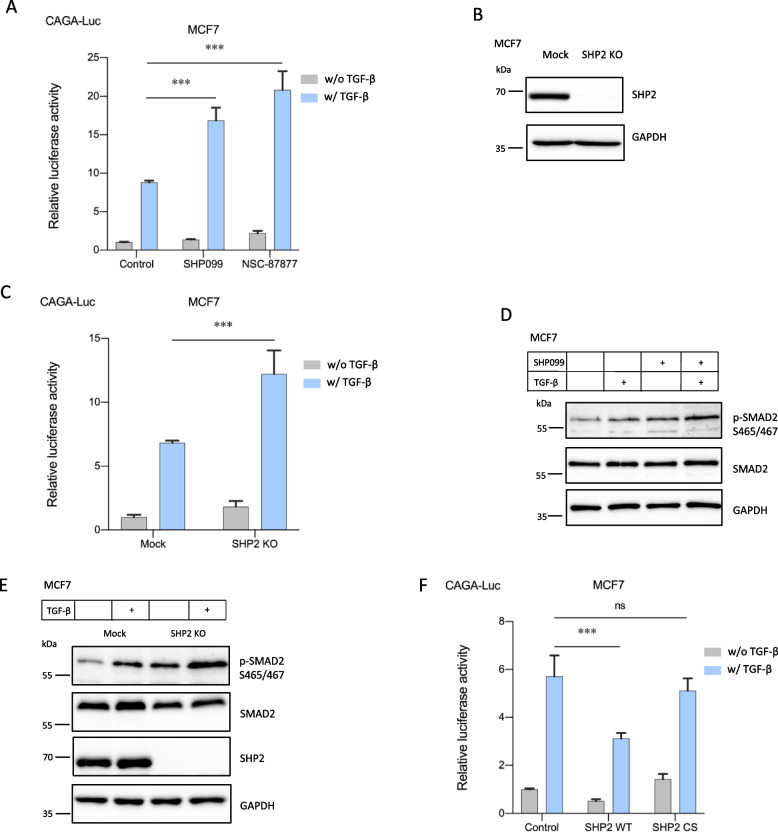
SHP2 inhibits TGF-β signaling.** A** MCF7 cells were transfected with CAGA-luciferase and β-galactosidase plasmids. Relative luciferase activities were measured after treatment or not with 10 μM SHP099, 10 μM NSC-87877 and TGF-β (5 ng/ml) for 2 days. Data are presented as mean ± SD (*n =* 3), analyzed by two-way ANOVA; *P < 0.05, **P < 0.01, ***P < 0.001. **B** Lysate of SHP2 knockout MCF7 cells was subjected to IB to verify knockout of SHP2. **C** Wild-type and SHP2 knockout MCF7 cells were transfected with CAGA-luciferase and β-galactosidase plasmids and stimulated with TGF-β (5 ng/ml) for 2 days; relative luciferase activities were measured. Data are presented as mean ± SD (*n =* 3), analyzed by two-way ANOVA; *P < 0.05, **P < 0.01, ***P < 0.001. **D** MCF7 cells were treated or not with 10 μM SHP099 and TGF-β (5 ng/ml) for 2 days. Cell lysates were prepared and subjected to IB with antibodies against SMAD2 and p-SMAD2. **E** Wild-type and SHP2 knockout MCF7 cells were treated with TGF-β (5 ng/ml) for 2 days. Cell lysates were prepared and subjected to IB with antibodies against SMAD2 and p-SMAD2. **F** MCF7 cells were transfected with wild-type RFP-SHP2 or RFP-SHP2 (C459S) together with CAGA-luciferase and β-galactosidase plasmids. Relative luciferase activities were measured after TGF-β (5 ng/ml) treatment for 2 days. Data are presented as mean ± SD (*n =* 3), analyzed by two-way ANOVA; *P < 0.05, **P < 0.01, ***P < 0.001

To investigate the role of SHP2 in TGF-β signaling, we employed the SMAD-responsive CAGA-luciferase reporter assay in MCF7 cells. We found that inhibiting SHP2 with the SHP1/2 phosphatase inhibitor NSC-87877 or the SHP2-specific allosteric inhibitor SHP099 enhanced CAGA-luciferase activity in response to TGF-β stimulation (Fig. 4A). Moreover, in an MCF7 cell line in which SHP2 had been knocked out (Fig. 4B), the response of cells to TGF-β stimulation was enhanced (Fig. 4C). Furthermore, either SHP2 inhibition (Fig. 4D) or SHP2 knockout (Fig. 4E) led to increased TGF-β -induced phosphorylation of SMAD2. Conversely, ectopic expression of wild-type SHP2 in MCF7 cells diminished the TGF-β-induced CAGA-luciferase reporter activity; notably, overexpression of an enzymatically inactive SHP2 mutant (C459S) did not elicit this inhibitory effect (Fig. 4F). Collectively, these findings emphasize a negative feedback role of SHP2 in regulating TGF-β signaling.

### Inhibition of SHP2 enhances the growth inhibitory effect of TGF*-*β

**Fig. 5:**
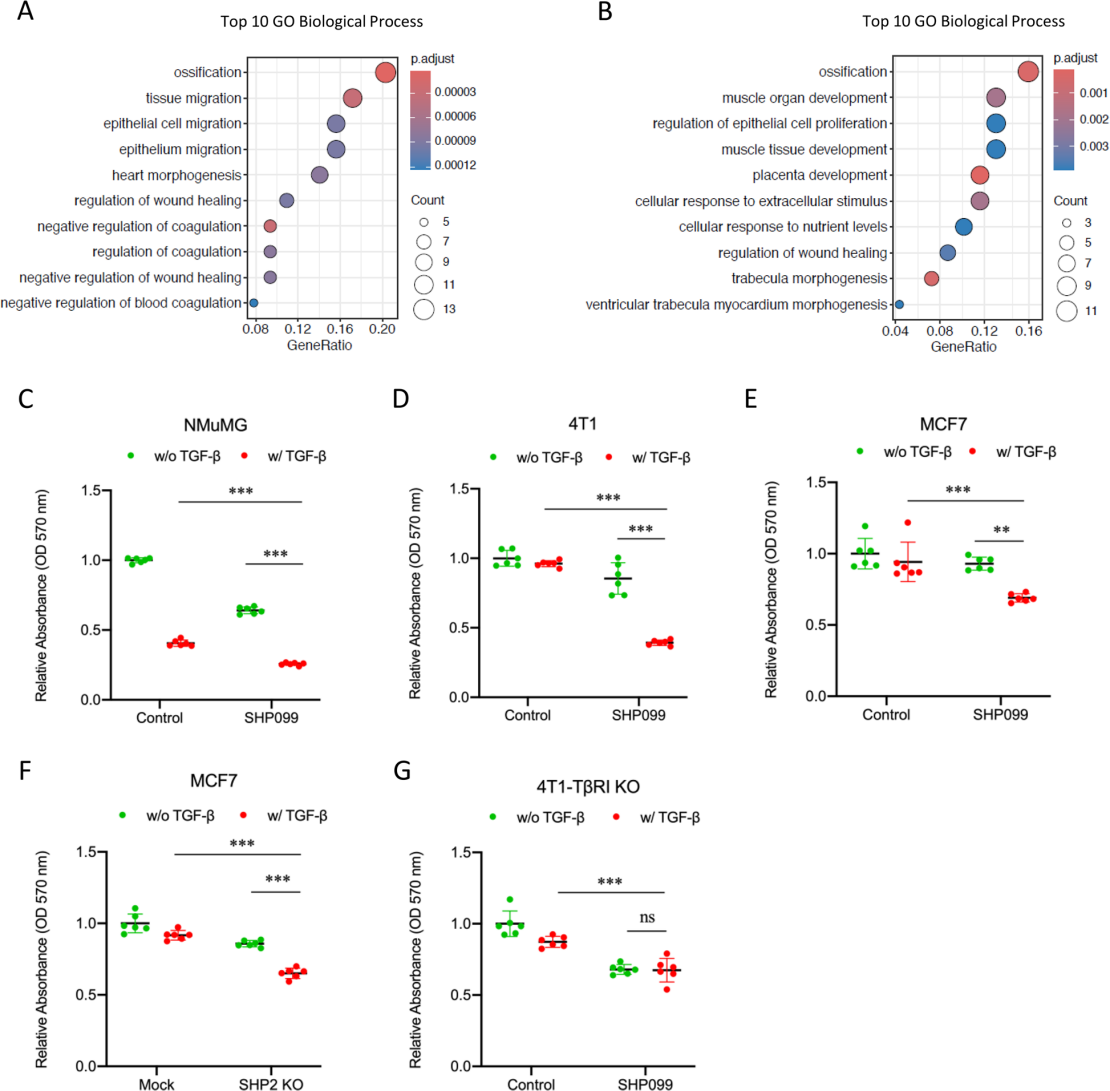
Inhibition of SHP2 enhances the growth inhibitory effect of TGF-β.** A**, **B** Gene ontology (GO) analysis of the top 10 biological processes of the regulated genes in wild-type (**A**) and SHP2 knockout (**B**) MCF7 cells, treated with TGF-β (5 ng/ml) compared with cells treated with 10 μM of the TbRI kinase inhibitor SB505124, as determined by RNA sequencing. **C**-**E** SHP2 was inhibited by treatment with 10 μM SHP099 in NMuMG (**C**), 4T1 (**D**) and MCF7 (**E**) cells; after treatment or not with TGF-β (5 ng/ml) for 3 days, cell amount was examined by MTT assay. Data are presented as mean ± SD (*n =* 6), analyzed by two-way ANOVA; *P < 0.05, **P < 0.01, ***P < 0.001. **F** Wild-type and SHP2 knockout MCF7 cells were treated with or without TGF-β (5 ng/ml) for 3 days, whereafter cell amount was examined by MTT assay. Data are presented as mean ± SD (*n =* 6), analyzed by two-way ANOVA; *P < 0.05, **P < 0.01, ***P < 0.001. **G** In 4T1 TβRI knockout cells, SHP2 was inhibited by treatment with 10 μM SHP099. After treatment or not with TGF-β (5 ng/ml) for 3 days, cell amount was examined by MTT assay. Data are presented as mean ± SD (*n =* 6), analyzed by two-way ANOVA; *P < 0.05, **P < 0.01, ***P < 0.001

To further explore the role of SHP2 in TGF-β-induced physiological responses, we performed RNA-Seq of MCF7 cells, before and after knockout of SHP2. Gene ontology (GO) analysis of differentially expressed genes revealed an enrichment of mRNAs related to the biological process of “epithelial cell migration”, as well as “tissue migration” and “epithelium migration” in wild-type MCF7 cells treated with TGF-β compared with cells treated with TβRI kinase inhibitor SB505124 (Fig. 5A). However, the GO biological processes switched to “regulation of epithelial cell proliferation”, as well as “muscle development” and “placenta development”, in SHP2 knockout cells treated with TGF-β compared to treatment with the TβRI kinase inhibitor (Fig. 5B), indicating that SHP2 knockout influenced the effect of TGF-β on cell proliferation. Consistently, pharmacological inhibition of SHP2 augmented TGF-β-mediated growth suppression in NMuMG cells and sensitized 4T1 and MCF7 cells to TGF-β-induced growth inhibition (Fig. 5C-E), and genetic ablation of SHP2 in MCF7 cells yielded similar results (Fig. 5F). In contrast, no effect of TGF-b-induced growth inhibition after SHP2 inhibition, was seen in 4T1 cells lacking TβRI (Fig. 5G). These findings suggest that SHP2 suppresses TGF-β-induced growth inhibition, and that this effect is dependent on TβRI.

### Inhibition of SHP2 enhances TGF*-*β*-*induced cell senescence

**Fig. 6:**
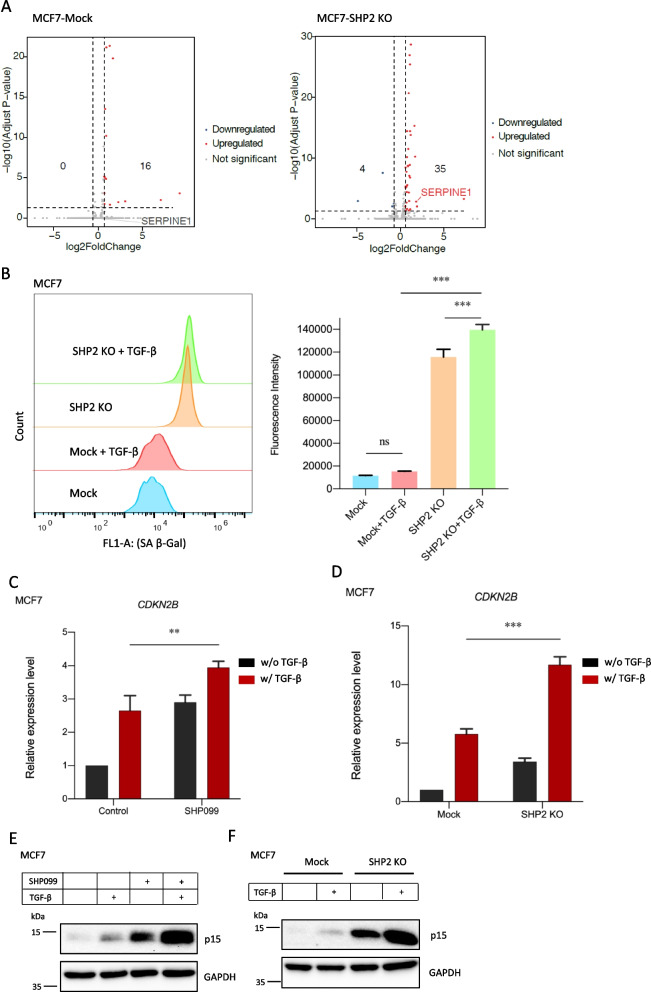
Inhibition of SHP2 enhances TGF-β-induced cell senescence. **A** Volcano plots of differentially regulated genes in response to TGF-β (5 ng/ml) treatment for 1.5 h in wild-type (left) and SHP2 knockout (right) MCF7 cells, as determined by RNA sequencing. Fold change thresholds were set to −1.5 and + 1.5. Upregulated genes are shown in red and downregulated genes are shown in blue. **B** Senescence of wild-type and SHP2 knockout MCF7 cells, treated or not with TGF-β (5 ng/ml) for 3 days, was determined by staining for SA b-Gal, and analyzed by flow cytometry (left panel) and statistical analysis (right panel). Data are presented as mean ± SD (*n =* 3), analyzed by two-way ANOVA; *P < 0.05, **P < 0.01, ***P < 0.001. **C** qPCR analysis of *CDKN2B* mRNA levels in MCF7 cells treated or not with 10 μM SHP099 and TGF-β (5 ng/ml) for 2 days. Data are presented as mean ± SD (*n =* 3), analyzed by two-way ANOVA; *P < 0.05, **P < 0.01, ***P < 0.001. **D** qPCR analysis of *CDKN2B* mRNA levels in wild-type and SHP2 knockout MCF7 cells treated with TGF-β (5 ng/ml) for 2 days. Data are presented as mean ± SD (*n =* 3), analyzed by two-way ANOVA; *P < 0.05, **P < 0.01, ***P < 0.001. **E** Lysates of MCF7 cells treated or not with 10 μM SHP099 and TGF-β (5 ng/ml) for 2 days, were subjected to IB with a p15 antibody. **F** Lysates of wild-type and SHP2 knockout MCF7 cells, treated or not with TGF-β (5 ng/ml) for 2 days, were subjected to IB with a p15 antibody

To explore the mechanism by which SHP2 influences TGF-β-induced growth inhibition, we employed RNA-Seq to compare the differently expressed genes in wild-type and SHP2 knockout MCF7 cells treated with TGF-β; *SERPINE1* was among the most upregulated genes in TGF-β stimulated SHP2 knockout cells, but not in wild-type cells (Fig. 6A). *SERPINE1* functions as a senescence marker, the product of which is secreted by senescent cells together with a plethora of factors that is collectively referred to senescent associated secretory phenotype (SASP) [4]. In wild-type cells, TGF-β did not induce significant senescence, while the loss of SHP2 induced senescence of MCF7 cells and sensitized cells to TGF-β-induced senescence, as corroborated by increased SA-β-gal staining in SHP2 knockout cells treated with TGF-β (Fig. 6B).

*CDKN2B* is a well-established transcriptional marker of senescence, the product of which (p15) promotes growth arrest induced by TGF-β [10, 35]. We found that TGF-β-induced upregulation of *CDKN2B* was further enhanced after treatment of the SHP2 inhibitor SHP099 (Fig. 6C). Similar results were observed in SHP2 knockout MCF7 cells (Fig. 6D). Consistently, the TGF-β-induced level of the *CDKN2B* product p15 was further increased by SHP2 inhibition (Fig. 6E) or knockout (Fig. 6F). These observations suggest that SHP2 inhibition positively regulates TGF-β-mediated growth arrest and cell senescence, through enhancing TGF-β-induced upregulation of p15 encoded by *CDKN2B*.

### Inhibition of SRC promotes TGF*-*β*-*induced senescence and growth inhibition in breast cancer cells

**Fig. 7:**
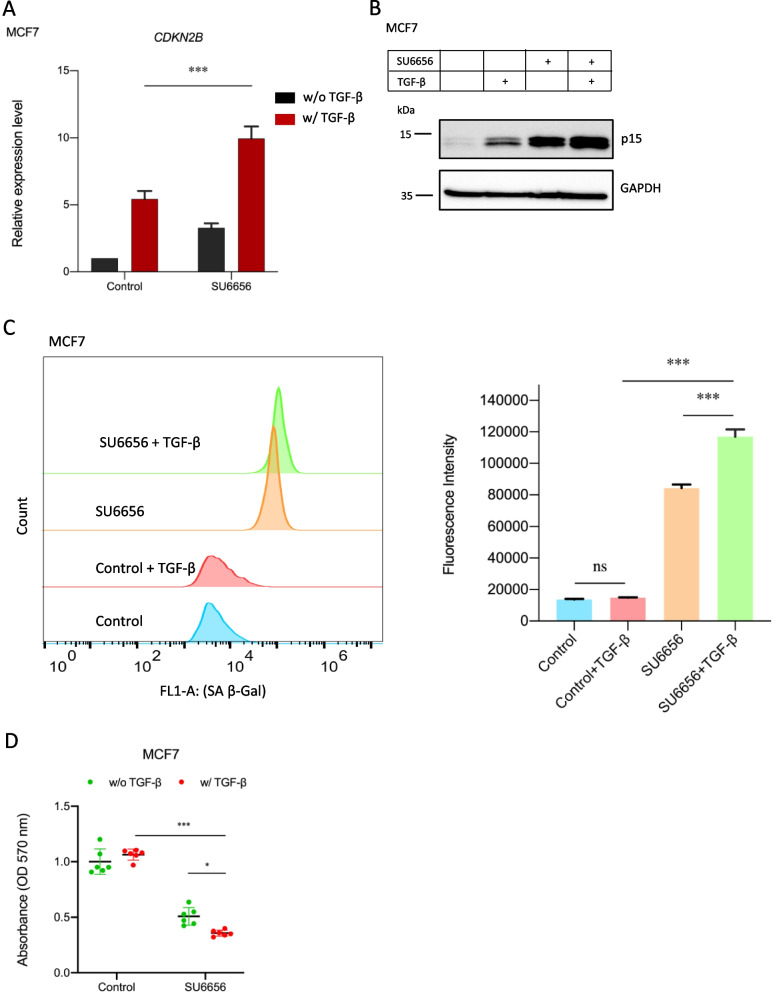
Inhibition of SRC promotes TGF-β-induced senescence and growth inhibition in breast cancer cells.** A** qPCR analysis of *CDKN2B* mRNA levels in MCF7 cells treated or not with 5 μM SU6656 and TGF-β (5 ng/ml) for 2 days. Data are presented as mean ± SD (*n =* 3), analyzed by two-way ANOVA; *P < 0.05, **P < 0.01, ***P < 0.001. **B** Lysates of MCF7 cells treated or not with 5 μM SU6656 and TGF-β (5 ng/ml) for 2 days were subjected to IB with a p15 antibody. **C** Senescence of MCF7 cells, treated or not with 5 μM SU6656 and TGF-β (5 ng/ml) for 3 days was determined by SA b-Gal staining and analyzed by flow cytometry (left panel) and statistical analysis (right panel). Data are presented as mean ± SD (*n =* 3), analyzed by two-way ANOVA; *P < 0.05, **P < 0.01, ***P < 0.001. **D** MCF7 cells were treated or not with 5 μM SU6656 and TGF-β (5 ng/ml) for 3 days. Cell number was assessed by MTT assay. Data are presented as mean ± SD (*n =* 6), analyzed by two-way ANOVA; *P < 0.05, **P < 0.01, ***P < 0.001

Since we found that SRC phosphorylates and activates SHP2, and that SHP2 inhibition enhances TGF-β-induced senescence and growth inhibition, we explored the role of SRC in TGF-β signaling. Treatment of cells with the SRC inhibitor SU6656 increased TGF-β-induced transcription of *CDKN2B* (Fig. 7A). Consistently, the TGF-β-induced level of the *CDKN2B* product p15 was further increased by SRC inhibition (Fig. 7B). Furthermore, SRC inhibition promoted TGF-b-induced senescence and growth inhibition (Fig. 7C, D). These results suggest that SRC inhibition sensitizes cells to TGF-β-induced senescence and growth inhibition, presumably by preventing activation of SHP2.

### SHP2 inhibition increasesthe stabilityof TβRI

**Fig. 8:**
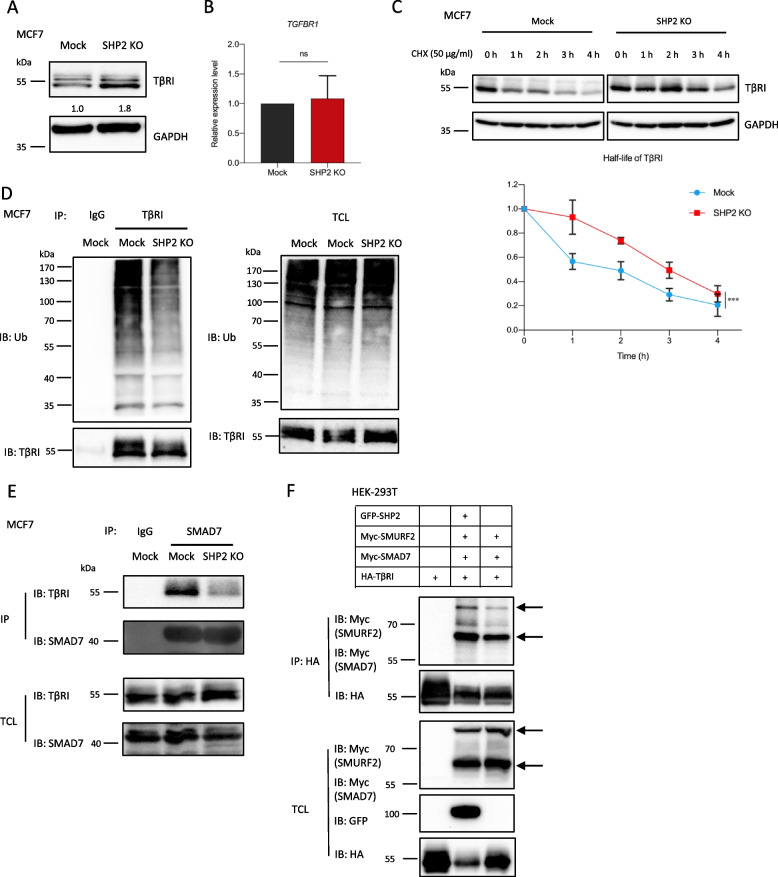
SHP2 inhibition increases the stability of TβRI. **A** Lysates of wild-type and SHP2 knockout MCF7 cells were subjected to IB with a TβRI antibody. **B** qPCR analysis of *TGFBRI* mRNA levels in wild-type and SHP2 knockout MCF7 cells. Data are presented as mean ± SD (*n =* 3), analyzed by Student’s *t*-test; *P < 0.05, **P < 0.01, ***P < 0.001. **C** Wild-type and SHP2 knockout MCF7 cells were treated with cycloheximide (CHX) for indicated time periods; lysates were then subjected to IB with a TβRI antibody (upper panel); the lower panel shows quantification of TβRI. Data are presented as mean ± SD (*n =* 3), analyzed by two-way ANOVA; *P < 0.05, **P < 0.01, ***P < 0.001. **D** Wild-type and SHP2 knockout MCF7 cells were pretreated with 10 μM MG132 for 8 h; cell lysates were then subjected to IP with a TβRI antibody, followed by IB with a ubiquitin (Ub) antibody. **E** Lysates of wild-type and SHP2 knockout MCF7 cells were subjected to IP with control IgG or a SMAD7 antibody, followed by IB with antibodies against TβRI and SMAD7. **F** Lysates of HEK-293 T cells transfected with indicated plasmids were subjected to IP with control a HA antibody, followed by IB with antibodies against Myc, GFP and HA

To elucidate the mechanism by which SHP2 modulates TGF-β signaling, we examined the levels of key components involved in TGF-β signal transduction in MCF7 cells, depleted or not of SHP2. Our analysis revealed a specific increase in TβRI expression upon SHP2 ablation (Fig. 8A). However, no significant alteration was observed of the *TGFBRI* mRNA level (Fig. 8B), suggesting that SHP2 influences TβRI stability. To investigate this possibility further, we performed a cycloheximide (CHX) chase assay, which demonstrated that SHP2 knockout extended the half-life of TRI (Fig. 8C), with a concomitant reduction of the overall ubiquitination level of TβRI (Fig. 8D). SMAD7 acts as a negative regulator in TGF-β signaling by recruiting SMURF2 to TβRI, promoting ubiquitination and proteasomal degradation [5]. We therefore investigated whether knockout of SHP2 influenced the interaction between SMAD7 and TβRI,a co-immunoprecipitation assay revealed that SHP2 knockout decreased the interaction between TβRI and SMAD7 in MCF7 cells (Fig. 8E). We then explored the effect of overexpression of SHP2 on the interaction between SMAD7, SMUFR2 and TβRI. As expected, overexpression of SHP2 increased the interaction between SMAD7 and TβRI, as well as the interaction between SMURF2 and TβRI (Fig. 8F). These results suggest that SHP2 knockout leads to a decreased ubiquitination-dependent degradation of the receptor.

**Fig. 9:**
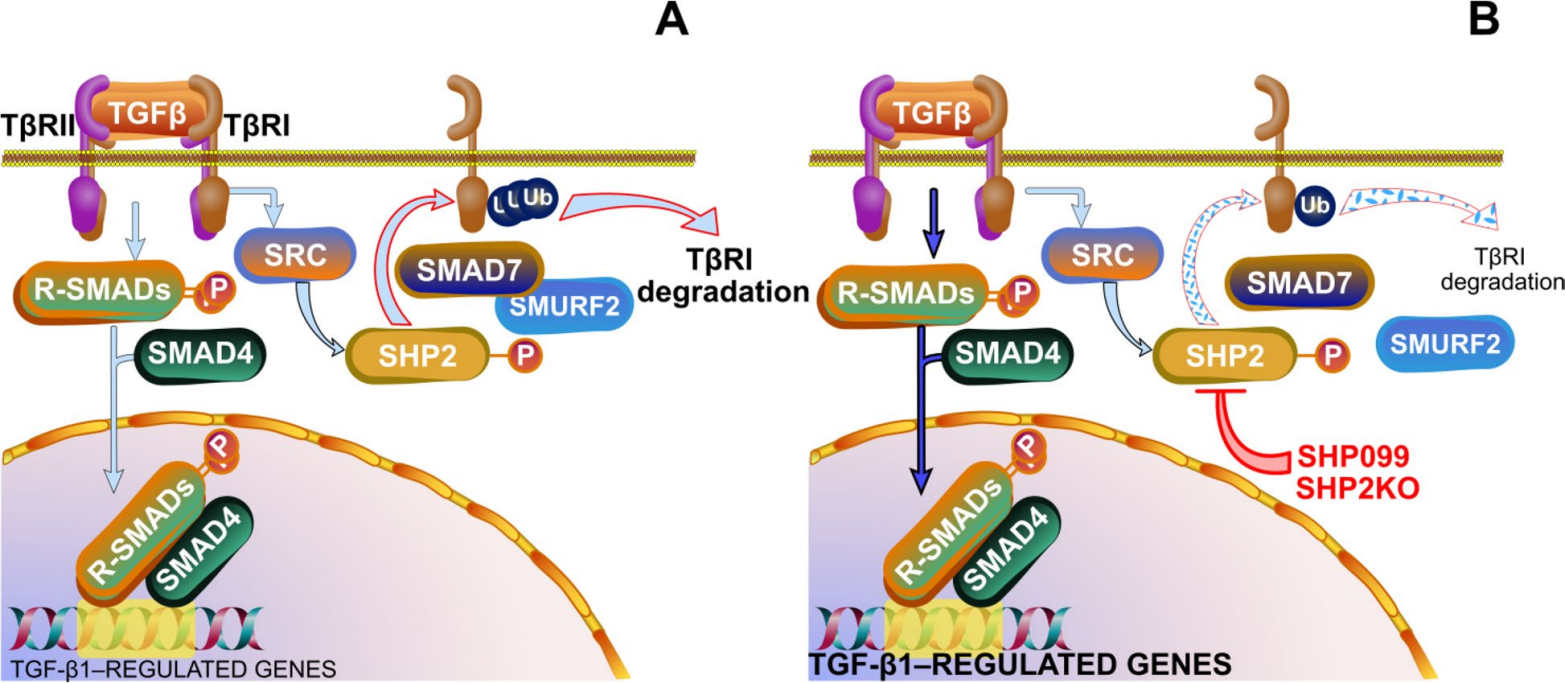
Proposed model for negative feedback regulation of TGF-β signaling by SRC and SHP2. **A** TGF-β stimulation promotes SRC-dependent phosphorylation and activation of SHP2 at Tyr542. Activated SHP2 interacts with TβRI and negatively regulates TGF-β signaling by decreasing receptor stability, associated with increased SMAD7 binding and receptor ubiquitination. Consistent with previous work, increased SMAD7 binding may facilitate recruitment of the E3 ubiquitin ligase SMURF2 and promote TGF-β receptor degradation [12]. This reduces SMAD2 activation and weakens TGF-β-induced transcriptional responses, including induction of *CDKN2B* and *SERPINE1*, thereby limiting growth inhibition and senescence. **B** Inhibition or loss of SHP2 stabilizes TβRI, enhances SMADs signaling, and strengthens TGF-β-induced cytostatic and senescence responses in epithelial and breast cancer cells

Based on these findings, we propose a model in which TGF-β activates SRC, which then phosphorylates and activates SHP2; in turn, SHP2 attenuates TGF-β signaling by destabilizing TβRI, thereby forming a negative feedback loop (Fig. 9).

## Discussion

TGF-β exhibits a context-dependent role in cell growth regulation. While it functions as a growth inhibitor in several cell types, such as epithelial, endothelial, and hematopoietic cells, tumor cells often develop mechanisms to circumvent the growth suppressive activity of TGF-β [19, 35]. For example, proteomic and phospho-proteomic analyses have revealed that Tβ RII depletion in keratinocytes results in the accumulation of kinases within the ERK1/2 MAPK pathway, promoting invasive growth [31]. In the present study, we have demonstrated that TGF-β induces a physical interaction between SRC and SHP2, leading to SHP2 phosphorylation and activation, resulting in attenuation of TGF-β-induced growth inhibition. Notably, pharmacological inhibition of SHP2 with the specific inhibitor SHP099 significantly enhanced TGF-β-mediated growth inhibition in breast cancer cells.

Protein tyrosine phosphatases (PTPs) are established regulators of TGF-β signaling. For example, PTPN2 has been shown to dephosphorylate SMAD4 at Tyr95, thereby restoring TGF-β-induced anti-proliferative activity by reversing phosphorylation by the receptor tyrosine kinase ALK [23, 34]. Moreover, TGF-β has been reported to enhance SHP1 phosphatase activity in an AKT- and SMAD3-dependent manner, leading to the inhibition of IFNγ-induced immune evasion in non-small cell lung cancer (NSCLC) [30]. The involvement of SHP2 in TGF-β signaling has also been documented in fibroblasts, where it regulates TGF-β-induced STAT3 signaling [33]. Recent work by Lai et al. demonstrated that SHP2 inhibition sustains TGF-β signaling by preventing SMURF2 dephosphorylation in bladder and lung cancer cells [13]. While these studies have elucidated a role of SHP2 in modulating TGF-β signaling, the reciprocal regulation of SHP2 by TGF-β and its potential role in feedback regulation have remained largely unexplored. In this study, we have shown that TGF-β induces SHP2 phosphorylation at Tyr542 in a manner dependent on the kinase activity of SRC, but not on that of TβRI. We moreover showed that TGF-β promotes the interaction between SRC and SHP2, and that pharmacological inhibition of SRC kinase activity inhibits TGF-β-induced SHP2 phosphorylation.

A precise control of TGF-β signaling is essential for maintaining physiological homeostasis. We found that SHP2 inhibition enhances TGF-β -induced SMAD2 phosphorylation, as well as transcriptional responses. Conversely, ectopic expression of wild-type SHP2 attenuated the response of cells to TGF-β stimulation, an effect not observed when a phosphatase-inactive SHP2 mutant was expressed. Interestingly, we found that SHP2 ablation resulted in increased TβRI protein levels, suggesting that SHP2 promotes TβRI degradation. Mechanistically, we demonstrated that SHP2 knockout reduced the binding of TβRI to SMAD7, a well-characterized negative regulator of TGF-β signaling which promotes TβRI degradation [1, 12]. A previous study has shown that SHP2 facilitates the interaction between SMAD7 and SMURF2 [13]. We have demonstrated that SHP2 facilitates the binding of TβRI to SMAD7 as well as to SMURF2 (Fig. 8F). Thus, we propose that SHP2 knockout reduces the interaction between TβRI and SMAD7 and causes less recruitment of SMURF2 to TβRI, leading to decreased ubiquitination and an increased stability of TβRI.

These findings also clarify the relationship between SRC-mediated tyrosine phosphorylation of TβRI and SHP2-dependent regulation of SMAD signaling. In our previous study, SRC activation and SRC-mediated phosphorylation of TβRI were mainly linked to non-canonical TGF-β responses, such as fibronectin production, actin reorganization and cell migration, whereas SRC inhibition had little effect on SMAD2 phosphorylation [28]. In the present study, we show that SHP2 inhibition increases SMAD2 phosphorylation primarily by stabilizing TβRI. Thus, SRC has two separable roles in TGF-β signaling,it contributes to non-canonical signaling responses and, via activation of SHP2, limits TGF-b signaling by promoting SMAD7-associated TβRI degradation. The precise function of individual SRC-phosphorylated tyrosine residues in TβRI remains to be determined.

TGF-β is known to induce a cytostatic effect through the upregulation of cyclin-dependent kinase (CDK) inhibitors, such as *CDKN1A*, *CDKN2A*, and *CDKN2B* [35], which are also key transcriptional markers of cellular senescence [10]. Previous studies have implicated SHP2 as a suppressor of senescence in glioblastoma and breast cancer [14, 25]. Consistent with these reports, our results demonstrate that SHP2 inhibition enhances TGF-β-mediated *CDKN2B* expression at both mRNA and protein levels. We also observed that SHP2 inhibition augmented TGF-β-induced inhibition of proliferation, as well as cellular senescence. These findings underscore a significant role of SHP2 in modulating TGF-β-mediated growth inhibition and senescence responses.

In parental 4T1 and MCF7 cells, SHP2 inhibition had only a limited effect on basal proliferation, but clearly enhanced the growth-inhibitory response to TGF-β. In contrast, in TβRI-knockout 4T1 cells, treatment with SHP099 reduced basal cell growth, yet it did not potentiate the effect of TGF-β. These findings suggest that the ability of SHP2 inhibition to augment TGF-β-induced growth suppression is dependent on TβRI, whereas the basal anti-proliferative effect of SHP2 inhibition is at least partly independent of TβRI. Thus, our findings support the notion that SHP2 both enhances the TβRI-dependent cytostatic effect of TGF-β, and in addition, affects additional pathways that regulate basal proliferation.

TGF-β has pleiotropic biological effects, depending on the types of cells and stage of disease. In the early stages of cancer, it suppresses the proliferation of cancer cells, however, it promotes cancer progression by inducing EMT and metastasis of cancer cells in the late stage of cancer. We have shown that SHP2 inhibition increases the response of cells to TGF-β, including TGF-β-induced growth inhibition. However, SHP2 knockout had less effect on EMT and migration (Fig. 5A, B). This may be due to the central role of SHP2 in many signaling pathways, including receptor tyrosine kinase (RTK) signaling pathways; knockout of SHP2 may thus both affect signaling pathways that promote and suppress EMT.

Taken together, our data support a model in which TGF-β-activated SRC phosphorylates SHP2 at Tyr542 and enhances its activity. Activated SHP2 then interacts with TβRI and functions as a negative regulator of TGF-β signaling by promoting receptor destabilization (Fig. 9). Mechanistically, our results suggest that SHP2 facilitates the association of SMAD7 with TβRI, leading to enhanced receptor ubiquitination and reduced receptor stability. Consequently, downstream SMAD2 phosphorylation and TGF-β-dependent transcriptional responses are attenuated. Functionally, this limits the induction of cytostatic and senescence-associated genes, including *CDKN2B* and *SERPINE1*, thereby reducing TGF-β-induced growth inhibition and senescence. Conversely, pharmacological inhibition or genetic ablation of SHP2 stabilizes TβRI, enhances SMAD signaling, and strengthens the cytostatic response to TGF-β. These findings identify SHP2 as part of a SRC-dependent negative feedback loop that restrains TGF-β tumor-suppressive signaling in epithelial and breast cancer cells.

A limitation of the present study is that the proposed SRC-SHP2-TβRI regulatory axis was characterized in cultured epithelial and breast cancer cell models. Although our biochemical, genetic, and functional data strongly support the mechanism, future studies in appropriate in vivo tumor models will be important to define the importance of this pathway in TGF-β signaling.

## Conclusions

Our observations provide insight into a mechanism for feedback control of TGF-b signaling, via SRC-mediated SHP2 phosphorylation, leading to destabilization of TbRI. We further demonstrate that SHP2 inhibition positively regulates TGF-β signaling by decreasing TβRI degradation through impeding the interaction between SMAD7 and TβRI, thereby enhancing growth arrest of cells, and the induction of senescence-associated genes, such as *SERPINE1* and *CDKN2B.*

## Data Availability

The RNA-seq data have not been uploaded to the public repository when submission. The RNA-seq data will be uploaded to NCBI after the acceptance of the manuscript.

## Abbreviations

SHP2: SRC homology 2-containing protein tyrosine phosphatase 2
TGF-β: Transforming growth factor β
co-IP: Co-immunoprecipitation
PI3K: Phosphatidylinositiol-3´-kinase
PTPN: Protein tyrosine phosphatase non-receptor
FGF: Fibroblast growth factor
PDGF: Platelet-derived growth factor
EMT: Epithelial-mesenchymal transition
IB: Immunoblotting
pNPP: *p*-Nitrophenyl phosphate
SASP: Senescent associated secretory phenotype
